# *In Vitro* Antimicrobial Activity of Spices and Medicinal Herbs against Selected Microbes Associated with Juices

**DOI:** 10.1155/2016/9015802

**Published:** 2016-01-04

**Authors:** Romika Dhiman, Neeraj Aggarwal, Kamal Rai Aneja, Manpreet Kaur

**Affiliations:** ^1^Department of Microbiology, Kurukshetra University, Kurukshetra, India; ^2^Vaidyanath Research, Training and Diagnostic Centre, Kurukshetra, India

## Abstract

In the present investigation, comparison of antimicrobial activities of different spices,* Curcuma longa*,* Zingiber officinale*, and* Mentha arvensis*, and medicinal herbs, such as* Withania somnifera*,* Rauvolfia serpentina*,* Emblica officinalis*,* Terminalia arjuna*, and* Centella asiatica*, was evaluated. Different extraction solvents (acetone, methanol, ethanol, and water) were used and extracts were examined against* Bacillus cereus*,* Serratia* sp.,* Rhodotorula mucilaginosa*,* Aspergillus flavus*, and* Penicillium citrinum* isolated from juices. Extracts from the medicinal herb and spices have significant activity.* B. cereus* was the most sensitive and* R. mucilaginosa* was the most resistant among the microorganisms tested. Ethanolic and methanolic extract of* C. asiatica* displayed maximum diameter of inhibition zone against bacteria and yeast and percentage mycelial inhibition against moulds. This study confirmed the potential of selected extracts of spices as effective natural food preservative in juices.

## 1. Introduction

Emergence of new technologies in food preservation leads to a reduction in the levels of preservatives and promotes the use of “naturally derived antimicrobials of animal, plants, and microbial origin [1]. Antimicrobial compounds derived from plants were used for centuries in food preservation. Egyptians, Chinese, and Indians used spices and essential oils since ancient time. Some of the spices such as mint, garlic, and ginger are still practiced in alternative health remedies in India [2, 3]. About 250 to 500 thousand plant species are estimated to exist on the planet and only between 1 and 10% of them are used as food by humans and other animals [4]. Spices and herbs used in foods as flavoring agents, in addition to enhancing flavors, were used as folk medicines and food preservatives. Spices and herbs extend the shelf life of foods by restricting rancidity through their antioxidant activity or through their bacteriostatic and bactericidal activity [5].

Spices, herbs, and their constituents are generally recogonised as safe (GRAS) and approved by several regulatory agencies such as US Food and Drug Act, the European Union standards, Codex Alimentarius, and Food Safety and Standards Authority of India [3]. In India, the trend of consumption of spices and herbs in food or using them as medicine aims to maintain proper sanitation, health, and hygiene and to increase longevity of life. Several spices such as ajowan, clove, ginger, black pepper, cumin, and asafetida are commonly used in the Indian diet [3, 6, 7]. Literature cited the work of several authors on the antimicrobial activity of plants against wide range of bacteria, yeasts, and moulds [2, 3, 8–20]. The general description of spices and herbs used in the present study is tabulated in Table 1.

Spices and herbs, owing to their natural origin, attract more attention of consumers that have doubt regarding the safety of chemical preservatives. Several plant extracts have gained momentum in recent years due to their bioactive principals and formed the basis of pharmaceutical and food processing industries [17, 21].

Unpasteurized fruit juice consumption has increased in the last decades, which is attributed to the contents of antioxidants, vitamins, and minerals. Fresh fruit juices are highly vulnerable to spoilage, since fluid components are in contact with air and microorganisms form the environment while handling [22]. Fruit juices spoilage bacteria include acid tolerant bacteria such as acetic acid bacteria, lactic acid bacteria,* Clostridium*,* Bacillus*, members of Enterobacteriaceae family (*Klebsiella* sp.,* Citrobacter* sp., and* Serratia* sp.), and some heat resistant bacteria such as* Alicyclobacillus acidoterrestris* and* Propionibacterium cyclohexinicum*. Among yeasts,* Pichia, Candida, Saccharomyces*, and* Rhodotorula* are commonly encountered genera responsible for spoilage of juices. Certain common moulds such as* Penicillium* sp.,* Aspergillus* sp.,* Eurotium*,* Alternaria*,* Cladosporium*,* Paecilomyces*, and* Botrytis* have also been reported in spoilage of fruit juices. The presence of pathogenic bacteria and mycotoxin producing mould cannot be ruled out in fruit juice which has been responsible for the increase in food borne outbreaks with the consumption of fresh fruit juices during the last two decades [14, 23, 24].

Therefore, the main objective of this study was to examine the* in vitro* antimicrobial activity of different spices and herbs extracts and to compare the effect of different solvents in the extraction method for antimicrobial activity.

## 2. Materials and Methods

### 2.1. Plant Materials

Three Indian spices, pudina, saunth, and haldi, were procured from local market in Yamunanagar, Haryana, India. Four medicinal plants herbs, amla, brahmi, ashwgandha, arjun, and sarpagandha were collected from Ch. Devi Lal Park at Khizrabad, Yamunanagar, Haryana, India. The taxonomic identity of these plants was confirmed by Dr. B. D. Vashishta, plant taxonomist, professor in the Department of Botany, Kurukshetra University, Kurukshetra. The scientific name and tested parts of the 8 plants are detailed in Table 1.

### 2.2. Extraction of Plant Material

Four different solvents, namely, ethanol, methanol, acetone, and aqueous (hot and cold), were used for extraction and plant extracts were prepared according to the methods described by Sharma et al. [25].

### 2.3. Test Microorganisms

In the previous study [24], microbiological analysis of fruit juices was done by serial dilution agar plate technique. On the basis of percentage of occurrence of microorganisms in juice samples, one Gram-positive bacterium, one Gram-negative bacterium, one yeast, and two moulds were selected for examining the antimicrobial activity of spices. Bacterial strains were identified on the basis of gram staining and biochemical and molecular characteristics (16S rRNA sequencing) [23]. Yeast was identified on the basis of staining, morphological, cultural characteristics, and molecular characteristics (28S rRNA sequencing). Moulds were identified on the basis of morphological and cultural characteristics and further identification was confirmed by CABI International Mycological Institute, UK.

Two bacteria, namely,* Serratia *(KC67407^*∗*^) and* Bacillus cereus* KRC1 (KC67408), one yeast,* Rhodotorula mucilaginosa* (KC67409), and two moulds,* Aspergillus flavus* (504472^*∗∗*^) and* Penicillium citrinum* (504473), were identified. The bacterial isolates were subcultured on nutrient agar and* R. mucilaginosa, A. flavus*, and* P. citrinum* were subcultured on potato dextrose agar and incubated aerobically at 37°C and 25°C, respectively. The media were procured from Hi Media Laboratory Pvt. Ltd., Bombay, India (^*∗*^nucleotide sequence of all microorganisms has been submitted to GenBank database which provided the GenBank accession number, KC67407–KC67409;  ^*∗∗*^International Mycological Institute reference number).

### 2.4. Screening for Antimicrobial Activity against Bacteria and Yeast

The acetone, methanol, ethanol, and hot and cold aqueous extracts of different plants were used for evaluation of antimicrobial activity by the agar well diffusion method. In this method, a pure isolate of bacteria and yeast was grown on NA and PDA plates and incubated at 37°C and 25°C for 24 h and 72 h, respectively. One plate of each microorganism was taken and colonies were transferred into normal saline (0.85%) under aseptic conditions. Density of each microbial suspension was adjusted to be equal to that of 10^6^ cfu/mL (standardized by 0.5 McFarland standard) and to be used as the inoculum for performing an agar well diffusion assay. 100 *μ*L of the inoculum of each test organism was spread onto the agar plates so as to achieve a confluent growth. The agar plates were allowed to dry and 8 mm wells were made with a sterile borer in the inoculated agar plates. The lower portion of each well was sealed with molten agar medium. The dried extracts were reconstituted to 20% in dimethyl sulfoxide (DMSO) to the final concentration of 100 mg/mL for the bioassay analysis. A 100 *μ*L volume of each extract was propelled directly into the wells (in triplicate) of the inoculated agar plates for each test organism. The plates were allowed to stand for 1 h at room temperature (40°C) for diffusion of the extract into agar and incubated at 37°C and 25°C for 24 h and 72 h, respectively. Sodium benzoate (100 mg/mL) was used as positive reference standards to determine the sensitivity of each microbial species tested. Sterile DMSO served as the negative control. The antimicrobial activity, indicated by an inhibition zone surrounding the well containing the extract, was recorded if the zone was greater than 8 mm. The experiments were performed in triplicate and the mean values of the diameter of inhibition zones ± standard deviations were calculated [34].

### 2.5. Screening for Antimicrobial Activity against Moulds

The antimould activity of plant extracts in different solvent was accessed by poison food technique. 100 *μ*L of plant extract with concentration of 100 mg/mL was poured into sterile Petri plate (90 mm diameter) and 15 mL of molten potato dextrose agar (PDA) was added to the Petri plate and swirled to achieve a uniform mixture and allowed them to solidify at room temperature. The solidified agar plates were inoculated at the centre with fungal disc (6 mm diameter), obtained from the actively growing one-week-old colony of the test fungus, placed with inoculums side down in the centre of each Petri plate aseptically and incubated at point 25°C for 7 days. DMSO was used as negative control and chemical preservative sodium benzoate served as the positive control. The antifungal activity of each extract was evaluated by measuring the radial growth of fungus in terms of diameter and expressed as percentage mycelia inhibition determined by applying the following formula [35–37]:

(1) $$\begin{array}{c} inhibition\;\;of\;\;mycelia\;\;growth\%=\frac{\left(d_{c}-d_{t}\right)}{d_{c}}\times 100, \end{array}$$

where *d*_*c*_ is average diameter of fungal colony in negative control plates and *d*_*t*_ is average diameter of fungal colony in extract added plates.

### 2.6. Determination of Minimum Inhibitory Concentration

Minimum inhibitory concentration (MIC) for each test organism was determined by the broth macrodilution method [38].

### 2.7. Statistical Analysis

The experimental results were repeated thrice in triplicate each time and expressed as mean ± SD and results were statistically evaluated using SPSS software version 16 at 5% significant level. Means were compared using Tukey's simultaneous test set at *p* < 0.05.

## 3. Results and Discussion

### 3.1. Antimicrobial Activity of Plant Extracts

The dietary herb and spices are used as food additives in foods not only to improve the sensory characteristics of food but also to increase the shelf life by reducing or eliminating survival of pathogenic bacteria [2].

In the present study, the antimicrobial activity of the different plant extracts in different solvents was examined. Perusal data in Table 2 revealed the mean diameters of the inhibition zones of all plant extracts against two bacteria and yeast and percentage mycelia inhibition against two moulds. There was significant variation (*p* < 0.05) observed between acetone, methanol, ethanol, cold aqueous, and hot aqueous solvents for the antimicrobial activities of each of the tested plant extracts and microorganisms.

For* B. cereus* (KC67408), the DIZ values of 18 extracts (accounting for 45% of the 40 tested extracts) were between 19.6 mm and 29.3 mm and those of 16 extracts (40%) were between 12.3 mm and 19.3 mm. However, 6 extracts had no inhibitory activity. Ethanolic extract of* C. asiatica* exhibited the strongest antibacterial activity (DIZ = 29.3 mm), followed by methanolic extract (DIZ = 26.3 mm) and ethanolic extract of* R. serpentina* (DIZ = 25.3 mm) (Table 2).

For* Serratia* (KC67407), a total of 25 extracts (62.5%) exhibited inhibitory activity with DIZ values ranging from 11.3 mm to 22.6 mm, and 47 extracts had low activity. The remaining 15 extracts had no inhibitory activity (Table 2). The sample with strongest antimicrobial activity was methanolic extract of* T. arjuna* (DIZ = 22.6 mm), followed by acetonic extract of* R. serpentina* (21.3 mm).

For* R. mucilaginosa* (KC67409), the DIZ values of 9 extracts (22.5%) ranged from 13.3 mm to 25.3 mm (Table 2). Thirty-one extracts (77.5%) showed no inhibitory activity. Ethanolic extract of* C. asiatica *showed the strongest inhibitory activity (DIZ = 20.6 mm), followed by methanolic extract of* T. arjuna* (19.3 mm).

Of the two moulds tested, for* A. flavus*, the percentage mycelial inhibition of 8 extracts (20%) varied from 20.6 mm to 26.3 mm. Nine extracts (22.5%) had low inhibitory activity (percentage mycelial inhibition ranging between 11.6 mm and 19.3 mm). The other 23 extracts (57.5%) showed no inhibitory activity. Ethanolic extract of* C. asiatica* exhibited the strongest antimould activity (percentage mycelial inhibition = 26.3 mm), followed by methanolic extract of* C. asiatica* and* T. arjuna* (24.6 mm). For* P. citrinum*, 8 extracts exhibited high inhibitory activity (percentage mycelial inhibition = 19.6 mm–24.3 mm) and nine extracts possessed less inhibitory activity (percentage mycelial inhibition ranging between 10.3 mm and 19.3 mm), while the other 23 extracts had no inhibitory activity. Methanolic extract of* C. asiatica* (24.3 mm) showed the strongest inhibitory activity, followed by ethanolic extract of* C. asiatica* and* W. somnifera* (23.3 mm).

Literature search revealed the* in vitro* and* in vivo* antimicrobial activities of plant extracts and essential oils against food borne pathogens but they are difficult to compare owing to the use of different methods of extraction, solvents, microbial strains, and antimicrobial test methods [3, 5, 16, 17, 39, 40]. In the present analysis, 4 plants,* C. asiatica*,* E. officinalis*,* M. arvensis*, and* T. arjuna*, exhibited broad spectrum activity against tested microbes. The present results were in line with the observation of earlier workers [5–7, 15–17, 41, 42]. Methanolic and ethanolic extract of* C. asiatica* exhibited maximum zone of inhibition against all tested microorganisms in the present investigation. The antimicrobial potential of* C. asiatica* was observed by Arumugam et al. [43] in a study to check the antimicrobial efficacy of* C. asiatica* against* B. cereus*,* S. aureus*, and* Pseudomonas aeruginosa* in four different solvents such as methanol, chloroform, water, and acetone and they found that methanolic extract of* C. asiatica* leaves at the concentration of 50 *μ*g per mL displayed activity (24–27 mm) against all tested microorganisms. The antimicrobial efficacy of* C. asiatica* is due to the presence of terpenoids [44]. Acetonic extracts of* T. arjuna* displayed maximum zone of inhibition (16–28 mm) against* S*. aureus,* Acinetobacter*,* P. aeruginosa*, and* Proteus mirabilis* in comparison to the other extracts (methanolic, ethanolic, and water extracts); however, all the extracts lacked activity against* Candida albicans* [32]. In the present investigation, methanolic and ethanolic extracts of* T. arjuna* exhibited antimicrobial activity against all tested microbes due to the presence of flavonoids (luteolin) [45, 46].

Ethanolic extract of* E. officinalis* leaves showed more activity against* P. aeruginosa*,* Proteus mirabilis*,* S. aureus*, and* B. cereus* in comparison to ethanolic extract of* M. arvensis* leaves at the concentration of 100 mg/mL [47]. However, in the present investigation, the ethanolic extracts of* M. arvensis* displayed more activity in comparison to* E. officinalis* ethanolic extract at the concentration of 100 mg/mL. The antimicrobial potential of* E*.* officinalis* and* M*.* arvensis *is attributed to the presence of hydrolysable tannins and menthol, respectively [48]. Although, in present investigation,* R. serpentina* and* W. somnifera* revealed antibacterial and antimould activity but lacked antiyeast activity, several authors confirmed antibacterial and antifungal potential of* W. somnifera* and* R. serpentina* [31, 33, 49, 50].

Sunilson et al. [15] observed the antimicrobial activity of* C. longa* and* Z. officinalis* in four different solvents (petroleum ether, chloroform, methanol, and water) against food borne pathogens. The solvent extracts of* C. longa* and* Z. officinalis* displayed antibacterial and antiyeast activity; however, in present investigation,* C. longa* and* Z. officinalis* possessed antibacterial activity.

The extraction of biologically active compound from plant material is largely dependent on the type of the solvent used in the extraction procedure. The present study revealed that the organic extracts provided more powerful antimicrobial activity compared to aqueous extracts. Among the organic extracts, alcoholic extracts displayed the best antimicrobial activity in comparison to acetonic extracts (Table 2), thus substituting the findings of earlier workers who rated methanol and ethanol as best solvent for the extraction of antimicrobial compounds from plants followed by acetone and water [5, 15, 16, 25]. Several compounds, tannins, flavonoids, coumarins, thiosulfinates, glucosinolates, and saponins, isolated from these plants are secondary metabolites which are responsible for the antimicrobial and medicinal properties of plants [2, 3, 8, 17].

### 3.2. Sensitivity of Five Tested Microbes

*B. cereus*,* Serratia*,* R. mucilaginosa*,* A. flavus*, and* P. citrinum* revealed varying sensitivities to the 40 tested extracts (Table 2). With respect to* B. cereus*, thirty-four extracts showed inhibitory activity and 16 extracts had no activity. In case of* Serratia*, 25 extracts displayed inhibitory activity and 15 extracts showed no activity.* R. mucilaginosa* was the most resistant of the tested microorganisms. Nine extracts exhibited inhibitory activity and 31 extracts had no inhibitory activity. Seventeen extracts showed inhibitory activity against two moulds (*A. flavus* and* P. citrinum*). Based on the inhibitory activity of plant extract, Gram-positive bacteria* B. cereus* demonstrated more sensitivity to the extracts than Gram-negative bacteria and fungi. The present findings are consistent with the results obtained by earlier workers who reported that plant extracts are more active against Gram-positive bacteria than against Gram-negative bacteria [3, 5, 16, 17, 48, 51–53]. This is attributed to the differences in the outer layers of Gram-negative and Gram-positive bacteria. Gram-negative bacteria possess an outer membrane and a unique periplasmic space not found in Gram-positive bacteria [5, 54, 55].

### 3.3. Determination of Minimum Inhibitory Concentration

The minimum inhibitory concentration was determined for 31 active plant extracts which show antimicrobial activity against microbes associated with juices (Table 3). The MIC of the 31 active plant extracts showed that MIC ranged between 3.12 mg/mL and 50 mg/mL for the organic extracts and between 25 and 50 mg/mL for the aqueous extracts. When comparing the MIC values of the tested plants, the ethanolic extract of* C. asiatica* appeared to be the most effective with the lowest inhibitory concentration being 3.15 mg/mL against* B. cereus*. Methanolic extracts of* C. asiatica*,* M. arvensis*, and* R. serpentina* were the second most effective plant extracts with MIC of 6.25 mg/mL. Many plant extracts show MIC in the range of 25–50 mg/mL and were thus shown to be less effective against microbes isolated from juices. It has been established that MIC results do not always correlate well with the DIZ values but, in present investigation, the observation of MIC related with the DIZ value might be due to the adoption of disc diffusion assay for both antimicrobial activity and MIC determination of plant extracts [16]. Similar results of MIC of different plants in different solvents have been observed by several authors against different microbes [3, 5, 9–13, 16, 56, 57].

## 4. Conclusion

The results of present work established that all the tested plant extracts possess antimicrobial activity against selected microbes associated with juices. Alcoholic extracts of medicinal herbs such as* C. asiatica, T. arjuna*, and* R. serpentina* displayed better antimicrobial activity than chemical preservative sodium benzoate; therefore these plant extracts have the potential to extend the shelf life or they are used as natural preservatives in fruit juices.

## Figures and Tables

**Table 1:** Ethnobotanical description, phytochemical composition, regulatory status, and part of plants used in antimicrobial study.

Scientific name	Common name	Family	Plant part tested	Phytoconstituents of part used	Traditional uses	References
*Centella asiatica *L.	*Jalbrahmi, mandukparni*	Apiaceae	Whole plant	Saponins, asiaticosides, brahminoside, and centelloside	Recommended for the treatment of various skin conditions such as leprosy, lupus, varicose ulcers, eczema, psoriasis, diarrhoea, fever, and amenorrhea and diseases of the female genitourinary tract	[26, 27]

*Curcuma longa*	Haldi	Zingiberaceae	Rhizome	Curcumin (diferuloylmethane), *α*-phellandrene, sabinene, borneol, zingiberene, and sesquiterpenes	Used to treat gastrointestinal upsets and arthritis pain and is tonic for the digestive system	[28]

*Emblica officinalis*	Amla	Euphorbiaceae	Leaves	Gallic acid, ethyl gallate, 1,2,3,4,6-penta-O-galloylglucose, and luteolin-4′-O-neohesperidoside	Source of Vitamin C, enhances food absorption, balances stomach acids, fortifies the liver, supports the heart, and promotes healthier hair	[29]

*Mentha arvensis*	Pudina	Lamiaceae	Leaves	Tannins, phenols, steroids, flavonoids, and volatile oils	Used to treat liver and spleen diseases, Asthma, and Jaundice	[30]

*Rauvolfia serpentina* Benth	Sarpagandha	Apocynaceae	Leaves	Alkaloids	Therapeutic actions being mainly effective in the treatment of hypertension and psychotic disorders like schizophrenia, anxiety, insomnia, and insanity	[31]

*Terminalia arjuna*	Arjun	Combretaceae	Leaves	Flavonoid	Used as a remedy for the treatment of ear ache	[32]

*Withania somnifera*	Ashwagandha	Solanaceae	Leaves	Steroidal withanolides	Claimed to have potent aphrodisiac rejuvenative and life-prolonging properties	[33]

*Zingiber officinale *	Saunth, dried ginger	Zingiberaceae	Rhizome	Gingerol (5-hydroxy-1-(4 hydroxy-3-methoxyphenyl) decan-3-one)	Commonly used in food products and beverages, carminative, antispasmodic, digestive, stomachic, vasodilator, appetizer, expectorant, bronchodilator, topical and local stimulant, analgesic, antiflatulent, aphrodisiac, antitussive, arthritis, rheumatism, sprains, muscular aches, pains, and laxative	[15]

**Table 2:** Antimicrobial activity of plant extracts in different solvents.

Plant	Solvent	Diameter of inhibition zone			Percentage mycelia inhibition	
		*B*. *cereus*	*Serratia *sp.	*R*. *mucilaginosa*	*A*. *flavus*	*P*. *citrinum*
*Centella asiatica*	Acetone	23.3_bx_^*∗*^ ± 1.52^*∗∗*^	—	—	—	—
	Methanol	27.3_cx_ ± 0.57	15.3_cy_ ± 0.57	18.3_cz_ ± 0.57	24.6_bu_ ± 0.57	23.3_bv_ ± 1.52
	Ethanol	29.3_dx_ ± 0.57	12.3_dy_ ± 1.52	20.6_dz_ ± 0.57	26.3_cu_ ± 1.52	24.3_cv_ ± 0.57
	Cold aqueous	—	—	—	—	—
	Hot aqueous	—	—	—	—	—

*Curcuma longa*	Acetone	22.3_bx_ ± 1.52	13.3_by_ ± 1.52	—	—	—
	Methanol	20.3_cx_ ± 1.52	—	—	—	—
	Ethanol	17.3_dx_ ± 1.52	—	—	—	—
	Cold aqueous	—	—	—	—	—
	Hot aqueous	—	—	—	—	—

*Emblica officinalis*	Acetone	22.3_bx_ ± 0.57	19.3_by_ ± 0.57	18.6_bz_ ± 1.52	19.3_bu_ ± 1.52	20.6_bv_ ± 1.52
	Methanol	19.6_cx_ ± 1.52	14.3_cy_ ± 0.57	16.3_cz_ ± 0.57	17.6_cu_ ± 1.52	18.3_cv_ ± 0.57
	Ethanol	17.3_dx_ ± 0.57	15.3_dy_ ± 0.57	14.3_dz_ ± 0.57	12.6_du_ ± 0.57	13.6_dv_ ± 0.57
	Cold aqueous	14.6_ex_ ± 0.57	—	—	—	—
	Hot aqueous	12.6_fx_ ± 0.57	—	—	—	—

*Mentha arvensis*	Acetone	26.3_bx_ ± 0.57	19.3_by_ ± 1.52	—	22.3_bu_ ± 0.57	20.6_bv_ ± 0.57
	Methanol	23.3_cx_ ± 0.57	14.3_cy_ ± 0.57	—	17.6_cu_ ± 1.52	15.3_cv_ ± 1.52
	Ethanol	24.3_dx_ ± 0.57	16.6_dy_ ± 0.57	—	16.6_du_ ± 1.52	14.3_dv_ ± 0.57
	Cold aqueous	19.3_ex_ ± 0.57	11.3_ey_ ± 0.57	—	—	—
	Hot aqueous	15.6_fx_ ± 1.52	—	—	—	—

*Rauvolfia serpentina*	Acetone	22.3_bx_ ± 0.57	21.3_by_ ± 1.52	—	17.3_bu_ ± 1.52	19.3_bv_ ± 1.52
	Methanol	26.6_cx_ ± 0.57	18.6_cy_ ± 0.57	—	15.6_cu_ ± 1.52	12.6_cv_ ± 1.52
	Ethanol	25.6_dx_ ± 1.52	17.3_dy_ ± 0.57	—	—	—
	Cold aqueous	19.3_ex_ ± 0.57	14.6_ey_ ± 1.52	—	—	—
	Hot aqueous	17.6_fx_ ± 0.57	12.6_fy_ ± 0.57	—	—	—

*Terminalia arjuna*	Acetone	19.3_bx_ ± 0.57	18.6_by_ ± 0.57	17.6_bz_ ± 0.57	20.6_bu_ ± 1.52	17.3_bv_ ± 0.57
	Methanol	24.6_cx_ ± 0.57	22.6_cy_ ± 0.57	19.3_cz_ ± 0.57	24.6_cu_ ± 0.57	21.3_cv_ ± 1.52
	Ethanol	21.3_dx_ ± 0.57	19.3_dy_ ± 0.57	16.6_dz_ ± 0.57	21.6_du_ ± 1.52	15.6_dv_ ± 0.57
	Cold aqueous	15.6_ex_ ± 0.57	14.6_ey_ ± 0.57	13.3_ez_ ± 0.57	11.6_eu_ ± 1.52	10.3_ev_ ± 0.57
	Hot aqueous	12.3_fx_ ± 0.57	—	—	—	

*Withania somnifera*	Acetone	20.3_bx_ ± 0.57	18.6_by_ ± 0.57	—	18.3_bu_ ± 0.57	19.6_bv_ ± 1.52
	Methanol	15.6_cx_ ± 1.52	13.3_cy_ ± 0.57	—	20.6_cu_ ± 0.57	22.3_cv_ ± 0.57
	Ethanol	14.3_dx_ ± 1.52	12.3_dy_ ± 1.52	—	22.6_du_ ± 1.52	23.3_dv_ ± 0.57
	Cold aqueous	—	—	—	—	—
	Hot aqueous	—	—	—	—	—

*Zingiber officinale *	Acetone	23.3_bx_ ± 0.57	18.6_by_ ± 0.57	—	—	—
	Methanol	21.3_cx_ ± 1.52	13.6_cy_ ± 0.57	—	—	—
	Ethanol	19.3_dx_ ± 0.57	11.3_dz_ ± 0.57	—	—	—
	Cold aqueous	17.6_ex_ ± 0.57	—	—	—	—
	Hot aqueous	12.3_fx_ ± 0.57	—	—	—	—

Sodium benzoate		20.6_ax_ ± 0.57	16.6_ay_ ± 0.57	14.6_az_ ± 0.57	30.6 ± 0.57	26.3 ± 0.57

^*∗*^Values, including diameter of the well (8 mm), are means of three replicates. ^*∗∗*^Standard deviation within five extracts and control of the same spice with three different microorganisms tested different letters are significantly  (*p* < 0.05) different. —: no activity.

**Table 3:** MIC of plant extracts in different solvents.

Plant	Solvent	MIC value of different plant extracts				
		*B*. *cereus*	*Serratia *sp.	*R*. *mucilaginosa*	*A*. *flavus*	*P*. *citrinum*
*Centella asiatica*	Acetone	12.5	Nt	Nt	Nt	Nt
	Methanol	6.25	25	25	12.5	12.5
	Ethanol	3.12	Nt	12.5	6.25	12.5

*Curcuma longa*	Acetone	12.5	50	Nt	Nt	Nt
	Methanol	12.5	Nt	Nt	Nt	Nt
	Ethanol	25	Nt	Nt	Nt	Nt

*Emblica officinalis*	Acetone	12.5	25	25	25	12.5
	Methanol	12.5	50	25	25	25
	Ethanol	25	50	50	Nt	50
	Cold aqueous	50	Nt	Nt	Nt	Nt

*Mentha arvensis*	Acetone	6.25	25	Nt	12.5	12.5
	Methanol	12.5	50	Nt	25	25
	Ethanol	6.25	25	Nt	25	50
	Cold aqueous	50	Nt	Nt	Nt	Nt
	Hot aqueous	50	Nt	Nt	Nt	Nt

*Rauvolfia serpentina*	Acetone	25	12.5	Nt	25	25
	Methanol	12.5	25	Nt	50	Nt
	Ethanol	6.25	25	Nt	Nt	Nt
	Cold aqueous	25	50	Nt	Nt	Nt
	Hot aqueous	25	Nt	Nt	Nt	Nt

*Terminalia arjuna*	Acetone	25	25	25	25	25
	Methanol	12.5	12.5	50	12.5	12.5
	Ethanol	12.5	25	25	12.5	50
	Cold aqueous	25	50	50	Nt	Nt

*Withania somnifera*	Acetone	12.5	25	Nt	25	25
	Methanol	50	50	Nt	12.5	12.5
	Ethanol	50	Nt	Nt	12.5	12.5

*Zingiber officinale *	Acetone	12.5	25	Nt	Nt	Nt
	Methanol	12.5	50	Nt	Nt	Nt
	Ethanol	25	Nt	Nt	Nt	Nt
	Cold aqueous	25	Nt	Nt	Nt	Nt
	Hot aqueous	Nt	Nt	Nt	Nt	Nt

Sodium benzoate		12.5	25	50	12.5	12.5

Nt: not tested.
